# Osteosynthese bei proximaler Humerusfraktur: Platte, Doppelplatte und mehr

**DOI:** 10.1007/s00113-025-01574-x

**Published:** 2025-04-24

**Authors:** Karl J. Sander, Julia Sußiek, Mats Jonas Wiethölter, Michael J. Raschke, J. Christoph Katthagen

**Affiliations:** Klinik für Unfall‑, Hand- und Wiederherstellungschirurgie, Universitätsklinikum Münster, Waldeyer Str. 1, 48149 Münster, Deutschland

**Keywords:** Humeruskopf, Frakturfixierung, Schraubenaugmentation, Bone graft, Osteoporose, Humeral head, Fracture fixation, Screw augmentation, Bone graft, Osteoporosis

## Abstract

Die proximale Humerusfraktur (PHF) tritt aufgrund des demografischen Wandels immer häufiger im klinischen Alltag auf. Gleichzeitig ist die operative Versorgung aufgrund komplexer werdender morphologischer Frakturmerkmale, vor dem Hintergrund der abnehmenden Knochenqualität, häufiger indiziert und anspruchsvoller durchzuführen. Die osteosynthetische Versorgung mithilfe der winkelstabilen Plattenosteosynthese ist neben der inversen Frakturprothetik weiterhin die Hauptsäule der operativen Behandlung. Das Therapiekonzept ist individuell abhängig von den morphologischen Frakturcharakteristika, dem patientenspezifischen Risikoprofil und der Expertise des Operateurs. Bei jüngeren Patienten ist, wenn umsetzbar, ein osteosynthetisches Vorgehen zu präferieren. In den letzten Jahren werden vermehrt Osteosynthesetechniken kombiniert, um einen Humeruskopferhalt und eine ausreichende Stabilität zu erreichen. Das Risiko für Komplikationen wie sekundäre Dislokation und Schrauben-Cut-out ist durch eine verbesserte Indikationsstellung und Operationstechnik deutlich gesunken. Die Doppelplattenosteosynthese bietet als eines dieser Kombinationsverfahren durch eine zusätzliche von ventral aufgebrachte Platte vielversprechende Möglichkeiten bei komplexen Frakturen und hat sich zunehmend im klinischen Alltag etabliert. Zementaugmentation von Schrauben oder unterschiedliche Bone-Grafts sind zusätzliche Möglichkeiten, winkelstabile Plattenosteosynthesen zu ergänzen, damit auch bei osteoporotischem Knochen ein gutes Rekonstruktionsergebnis erzielt werden kann.

## Abstract (en)

Due to demographic changes, proximal humeral fractures (PHF) are becoming increasingly more frequent in routine clinical practice. At the same time, surgical treatment is more frequently indicated and more demanding due to increasingly more complex fracture morphology and declining bone quality. Osteosynthesis using locking plate fixation remains the mainstay of surgical treatment alongside reverse fracture arthroplasty. The treatment concept is individually dependent on the characteristics of the fracture morphology, the patient-specific risk profile and the expertise of the surgeon. In younger patients fracture fixation is preferable, when feasible. In recent years, combinations of different surgical techniques have been increasingly used to achieve humeral head preservation and sufficient stability. The risk of complications, such as secondary dislocation and screw cut-out has significantly decreased in recent years due to improved evaluation of the indications and surgical techniques. As one of these combined procedures, double plate fixation offers promising options for complex fractures due to an additional plate applied ventrally. This technique is increasingly becoming established in routine clinical practice. Cement augmentation of screws and different bone grafts are additional options to supplement locking plate osteosynthesis so that a good reconstruction result can also be achieved in osteoporotic bone.

Die proximale Humerusfraktur (PHF) ist mit einer jährlichen Inzidenz von 110 Fällen/100.000 Einwohnern eine der am häufigsten auftretenden Frakturen im hohen Alter in Deutschland. Aufgrund des demografischen Wandels zeigt sich bereits eine deutliche Steigerung der Inzidenz, die sich in den nächsten Jahren fortsetzen wird [17]. Mit dieser Zunahme wird aufgrund der im Alter abnehmenden Knochenqualität gleichzeitig der morphologische Frakturbefund komplexer, sodass eine operative Versorgung häufiger notwendig wird [1]. Die Frakturversorgung stellt daher, trotz aller Neuerungen der letzten Jahre, weiterhin eine Herausforderung dar. Neben den geriatrischen, meist weiblichen Patienten gibt es einen zweiten Häufigkeitsgipfel bei jüngeren, meist männlichen Patienten mit einer PHF im Rahmen eines Hochrasanztraumas.

Ziel dieser Arbeit ist es, einen Überblick über die osteosynthetische Versorgung der PHF zu geben und Entscheidungshilfen für die Wahl der optimalen Behandlung bereitzustellen.

## Indikation zur osteosynthetischen Versorgung

Die osteosynthetische Versorgung stellt neben der konservativen Therapie und der prothetischen Versorgung eines der drei etablierten Hauptbehandlungskonzepte der PHF dar. Nicht oder geringgradig dislozierte Frakturen gelten als primäre Indikation für eine konservative Therapie. Bei der Wahl der operativen Behandlungsmethode gibt es vorgeschlagene Therapiealgorithmen, die als Orientierung dienen können, wie z. B. von Spross et al. [29]. Strikt definierte und validierte Handlungsempfehlungen existieren aktuell noch nicht. Die Wahl des optimalen operativen Therapieverfahrens erfordert eine individuelle und differenzierte Analyse der morphologischen Situation unter den Gesichtspunkten der Rekonstruierbarkeit, der Durchblutung und der Knochenqualität.

Aufgrund der zunehmend komplexeren morphologischen Frakturbefunde hat sich, wenn im initialen Röntgenbild entsprechende Hinweise vorliegen, die Computertomographie als Ergänzung zur Planung der Frakturversorgung etabliert. In die Therapieplanung sollte zudem eine sorgfältige Berücksichtigung patientenspezifischer Risikofaktoren wie Vorerkrankungen, Begleitverletzungen und funktioneller Anspruch des Patienten einbezogen werden. Ebenfalls sollten die Expertise und Erfahrung des Operateurs Einfluss auf die Auswahl des Therapieverfahrens haben.

Die Osteosynthese als gelenkerhaltende Maßnahme ist bei jüngeren Patienten bevorzugt

**Abb. 1:**
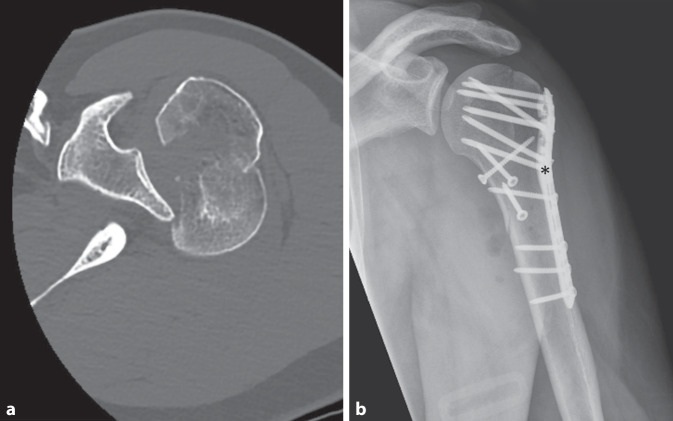
Aufnahmen eines 45-jährigen Patienten nach einem Verkehrsunfall mit posteriorer Luxationsfraktur Typ 5 nach Resch et al. [22]. **a** Präoperative Schicht der axialen CT-Bildgebung, **b** postoperative Röntgenbildgebung nach winkelstabiler Platten- und Schraubenosteosynthese. *Asteriskus* Kalkarschraube

Obwohl die Auswahl der Behandlungskonzepte nicht strikt definiert oder standardisiert ist, wird die Prothesenversorgung häufig bei älteren Patientengruppen mit höhergradigen Frakturen und geringer Knochenqualität als Therapie der Wahl eingesetzt. Die Osteosynthese als gelenkerhaltende Maßnahme ist insbesondere bei jüngeren Patienten bevorzugt [26]. Auch bei komplexen Frakturen, die klassische Indikationen für Frakturprothesen sind (z. B. Head-Split-Frakturen), besteht für junge Patienten die Möglichkeit des erfolgreichen Erhalts des Humeruskopfes (Abb. 1; [25, 26]). Zusätzlich ist aufgrund des bei jungen Patienten meist geringeren Operationsrisikos eine mögliche Folgeoperation wie eine Implantatentfernung oder arthroskopische Arthrolyse vertretbar. Diese Operationen sind regelmäßig nach der Versorgung von komplexen PHF notwendig und nicht als Komplikationen des operativen Vorgehens zu werten. Vielmehr sollten diese möglichen Folgeeingriffe bereits in der initialen Operationsaufklärung mit dem Patienten besprochen werden. In der Literatur werden Raten der Implantatentfernung nach PHF von ca. 20–25 % angegeben, wobei diese zentrumsspezifisch stark variieren [20].

Bei der Entscheidungsfindung für ein operatives Verfahren sollte zudem berücksichtigt werden, dass bei Versagen der Osteosynthese die Möglichkeit der sekundären Konversion auf eine Prothesenversorgung besteht. Eine primäre Prothesenimplantation stellt hingegen eine endgültige Versorgung dar.

Der Erhalt von Knochensubstanz sowie das Einheilen von Fragmenten mit adhärenten Weichteilen verbessern die Versorgungsqualität einer Prothesenimplantation, die bei pathologischen Folgeerscheinungen wie einer posttraumatischen Omarthrose erforderlich werden kann. Dies ist ein klarer Vorteil der osteosynthetischen Verfahren gerade bei jungen Patienten.

## Plattenosteosynthese

Nach wie vor wird die winkelstabile Plattenosteosynthese am häufigsten zur operativen Versorgung von PHF verwendet, jedoch geht der relative Anteil in Deutschland zurück [23]. Andere Verfahren wie die anatomische Frakturprothese haben derweil in der klinischen Praxis deutlich an Bedeutung verloren, während die Zahl der implantierten inversen Schulterprothesen vor dem Hintergrund der demografischen Alterung weiter steigt [10, 14].

Die operative Versorgung mithilfe einer Plattenosteosynthese erfolgt im eigenen Vorgehen über den deltoideopektoralen Zugang. Dieser bietet eine gute Übersicht und Möglichkeiten zur Frakturreposition. Alternativ kann der proximale Humerus auch über den transdeltoidalen lateralen „Delta-Split“-Zugang erreicht werden. Hilfreich ist die Lagerung des frakturierten Arms in einer intraoperativ bedienbaren Armhalterung, um Repositionsmanöver zu erleichtern.

Von Südkamp et al. wurde 2009 nach osteosynthetisch versorgter PHF eine Komplikationsrate bis zu 35 % festgestellt [30]. In den letzten Jahren hat sich diese nach der Plattenosteosynthese, wie 2016 von Haasters et al. publiziert, auf ca. 20 % reduziert. Dies ist neben Verbesserungen der Osteosynthesetechnik auch auf die Zunahme der inversen Schulterendoprothetik bei komplikationsgefährdeten Fällen in der Frakturversorgung zurückzuführen [7].

In die Operationsplanung ist der Aspekt der Humeruskopfdurchblutung einzubeziehen

Die avaskuläre Humeruskopfnekrose tritt in ca. 10–20 % der osteosynthetischen PHF-Versorgungen auf, wobei die Angaben zur Häufigkeit in der Literatur sehr stark variieren [20]. Um diese Komplikation zu vermeiden, sollten bereits bei der Operationsplanung die morphologischen Frakturmerkmale und die Dislokation der Fragmente vor dem Hintergrund der Humeruskopfdurchblutung evaluiert werden. Die Durchblutung des Humeruskopfes erfolgt hauptsächlich durch zwei der A. axillaris entspringende Gefäße. Die A. circumflexa humeri posterior verläuft gemeinsam mit dem N. axillaris durch die laterale Achsellücke. Von ventral verläuft die A. circumflexa humeri anterior um den Humeruskopf herum. Besonders der entlang des Sulcus intertubercularis verlaufende aufsteigende anterolaterale Ast ist bei der Präparation und Positionierung der lateralen Platte zu schonen [18].

Zu den typischen Frakturen mit kompromittierter Durchblutung gehören die Head-Split‑, Luxations-, 4‑Part-Frakturen und Frakturen im Bereich des Collum anatomicum. Große Dislokationen der Tubercula, ausgeprägte metaphysäre Trümmerzonen oder eine durch Dislokation verursachte Angulation des Kopfes gegenüber dem Schaft > 45° sind ebenfalls negative Prädiktoren für die Perfusion [12, 33].

Eine besondere Bedeutung für die Durchblutung und die Stabilität der Frakturversorgung stellt die mediale Kortikalis mit der inbegriffenen Kalkarregion dar, wie bereits Gardner et al. 2007 zeigen konnten [5]. Bei der osteosynthetischen Versorgung sollte besondere Aufmerksamkeit auf die Rekonstruktion und Stabilität dieser Region gelegt werden. Die mediale Säule zählt dann als stabil, wenn sie anatomisch reponiert ist, nicht frakturiert ist, der Kopf in den Schaft eingestaucht ist oder von lateral aufsteigende Kalkarschrauben eingebracht wurden. Die meisten modernen Platten verfügen über speziell ausgelegte Löcher zur Platzierung der Kalkarschrauben (Abb. 1b).

Für optimale funktionelle Ergebnisse nach der Plattenosteosynthese ist die anatomische Reposition unerlässlich. Besondere Aufmerksamkeit sollte auf die Dislokation und Angulation zwischen Humeruskopf und -schaft sowie auf eine mögliche Kranialisierung des Tuberculum majus durch den Zug der posterosuperioren Rotatorenmanschette gelegt werden. Schnetzke et al. zeigten in ihrer Studie, dass die Qualität der Reposition einen direkten Einfluss auf die postoperative klinische Funktion hat. Eine anatomische Reposition führt neben der Verbesserung des klinischen Outcome auch zu Reduktion der postoperativen Komplikationen. Durch das genaue Einpassen der Frakturfragmente kann das Risiko für eine sekundäre Dislokation mit einem resultierenden Cut-out der eingebrachten Schrauben gesenkt werden [27].

Neben der Reposition ist die korrekte Plattenlage ebenfalls entscheidend für das funktionelle Outcome. Die winkelstabile Platte wird 0,5–1 cm lateral des Sulcus intertubercularis aufgebracht. Die Plattenosteosynthese sollte zudem so positioniert werden, dass bei der Abduktion kein Impingement zwischen dem kranialen Plattenrand und dem Akromion entsteht. Die Befestigung der Rotatorenmanschettensehnen an der Platte mithilfe von Faden-Cerclagen hat sich bereits fest in der operativen Technik etabliert und schützt die Tubercula vor Dislokation durch Muskelzug.

Eine stabile Fixierung des Tuberculum majus ist für die Funktion des Schultergelenks unerlässlich

Das Tuberculum majus spielt eine Schlüsselrolle für die klinische Funktion nach der Rekonstruktion des Humeruskopfes. Daher ist bei der osteosynthetischen Versorgung besonders auf eine stabile Fixierung des Fragments zu achten. Eine sekundäre Dislokation des Fragments erfolgt in der Mehrzahl der Fälle posterosuperior und wird häufig durch eine avaskuläre Nekrose verursacht [21]. Im Fall einer solchen Komplikation ist die klinische Funktion durch den Verlust der Rotatorenmanschettenfunktion erheblich beeinträchtigt, sodass ein frühzeitiges operatives Eingreifen indiziert ist. Bei komplexen morphologischen Defekten des Tuberculum majus, wie beispielsweise mehrfragmentären Frakturen oder ausgeprägter Dislokation, gestaltet sich eine stabile Refixation mit den aktuell verfügbaren Implantaten jedoch schwierig. Derzeit werden Ansätze zur Weiterentwicklung bestehender Implantate entwickelt, um die Retention zu verbessern [24].

Bezüglich der postoperativen Funktion finden sich in der Literatur unterschiedliche Ergebnisse. Die systematische Übersichtsarbeit von Gupta et al. aus dem Jahr 2015 zeigt bessere funktionelle Ergebnisse nach offener Reposition und Fixierung (ORIF) gegenüber einer Versorgung mithilfe von Hemi- oder inverser Schulterprothese bei gleichzeitig höheren Komplikationsraten [6]. Neuere randomisierte und kontrollierte Studien belegen Vorteile im funktionellen Outcome auf der Seite der Versorgung mit einer inversen Prothese gegenüber der Plattenosteosynthese [4, 11]. Aufgrund der Vielzahl der osteoporotisch bedingten Frakturen ist die Literatur allerdings nicht aussagekräftig in Bezug auf ein junges Patientenkollektiv [3, 8]. Diesbezüglich sind weitergehende Untersuchungen notwendig.

## Doppelplattentechnik

In den letzten Jahren hat sich zunehmend die Doppelplattenosteosynthese etabliert, um auch bei kompromittierter Knochenqualität oder komplexen Frakturen kopferhaltend zu operieren. Hierbei wird neben der konventionellen, lateral aufliegenden winkelstabilen Platte eine weitere Platte, z. B. eine Drittelrohrplatte, von ventral befestigt [18, 32, 34].

**Abb. 2:**
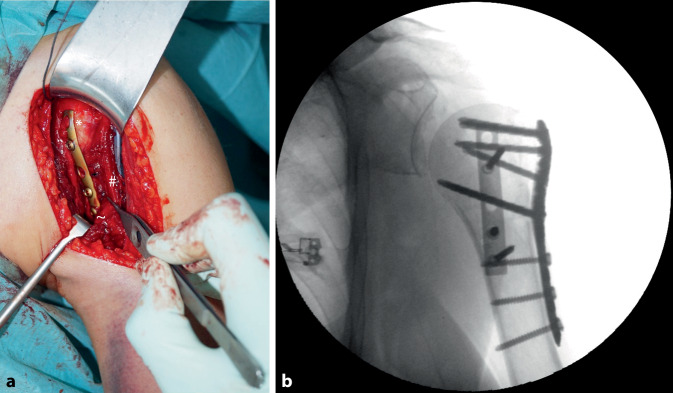
Bilder eines 78-jährigen Patienten. **a** Intraoperativer Situs mit angebogener Drittelrohrplatte. *Asteriskus* Tuberculum minus, *Pluszeichen* A./V. circumflexa humeri anterior, *Doppelkreuz* lange Bizepssehne, *Tilde* Ansatz des M. pectoralis major. **b** Intraoperative Röntgenbildgebung. (aus Michel et al. Double plating of proximal humeral fractures [18])

In unserem Vorgehen liegt die Platte kranial dem Ansatz des M. subscapularis am Tuberculum minus auf und verläuft medial des Sulcus intertubercularis bis zum distalen Ende unterhalb des M pectoralis major (Abb. 2).

Bei Frakturbeteiligung des Tuberculum minus kann eine additive Platte die Dislokation verhindern

Die Indikation für eine Doppelplattenosteosynthese nach einer PHF besteht u. a. bei verminderter Knochenqualität und komplexer morphologischer Fraktursituation. Im Fall einer Frakturbeteiligung des Tuberculum minus kann es zur Dislokation des Fragments durch den Muskelzug des M. subscapularis kommen; dies kann durch die zusätzliche Verankerung der additiven Platte verhindert werden.

Bei varisch abgekippten Frakturen liegt eine Beteiligung der medialen Säule vor. Die zweite eingebrachte Platte kann hier durch eine zusätzliche Rotationsstabilität und Abstützung ebenfalls einer sekundären Dislokation durch Abkippen nach ventral entgegenwirken. Ähnlich verhält es sich bei metaphysären Trümmerzonen oder Defektstrecken, wie biomechanisch durch unsere Arbeitsgruppe festgestellt werden konnte [16].

Biomechanische Veröffentlichungen der Arbeitsgruppe in Leipzig um Professor Hepp zeigten eine erhöhte Steifigkeit und größere Versagenslast der Doppelplattenosteosynthese [31, 34]. Klinisch beschreiben erste retrospektive Auswertungen von Warnhoff et al. und aus unserer Klinik vielversprechende Ergebnisse gerade bei jüngeren Patienten [19, 32]. Insgesamt ist die Literatur zur Doppelplattenosteosynthese aber noch begrenzt, und weitere Studien sind notwendig.

Ein mögliches Risiko der Doppelplattenosteosynthese gegenüber der etablierten Osteosyntheseverfahren ist die weitere Kompromittierung der durch die Fraktur und die laterale Platte bereits beeinträchtigten Humeruskopfperfusion. Die Rate der avaskulären Humeruskopfnekrose zeigte sich bei Warnhoff et al. erhöht bei ansonsten insgesamt vergleichbarer Komplikationsrate mit der Einzelplattenosteosynthese [32]. Aktuell ist unklar, ob dieser Anstieg kausal auf die Doppelplattenosteosynthese zurückzuführen oder durch eine erweiterte Indikationsstellung begründet ist. Hierfür sind weitere Untersuchungen und Daten erforderlich.

## Und mehr

**Abb. 3:**
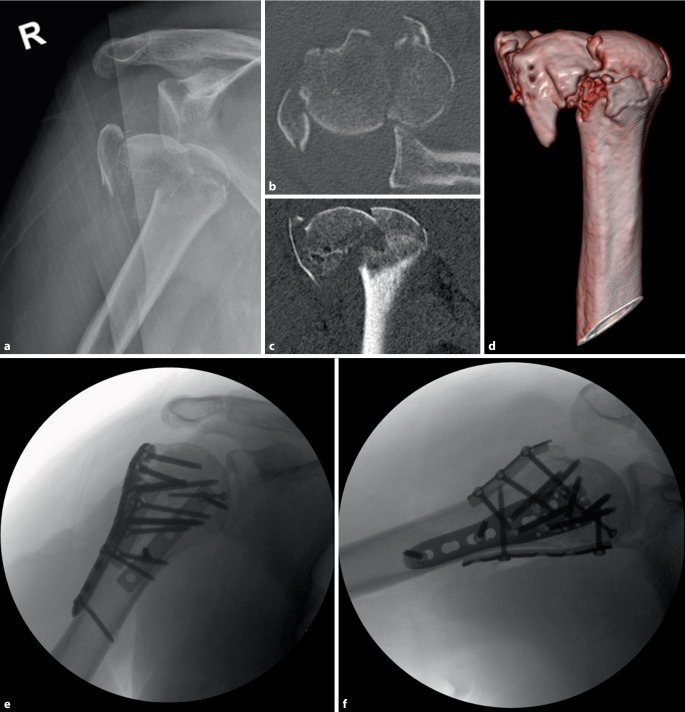
Aufnahmen eines 55-jährigen Patienten mit anteriorer Luxationsfraktur Typ 5 nach Resch et al. [22] nach einem Treppensturz. **a** Präoperative Röntgenbildgebung, **b** axiale Schicht der CT-Bildgebung, **c** koronare Schicht der CT-Bildgebung, **d** Volumenrekonstruktion der proximalen Humerus, **e** intraoperative a.-p.-Röntgenaufnahme, **f** intraoperative axiale Röntgenaufnahme

Neben der Platten- und Doppelplattenosteosynthese finden in der Frakturversorgung am proximalen Humerus weitere Verfahren als alleinige Versorgung oder in Kombination mit der Plattenosteosynthese Anwendung. Aufgrund der komplexeren morphologischen Fraktursituation werden individualisierte Versorgungen mit zwei oder mehr Osteosyntheseverfahren in Deutschland häufiger, wie Daten der Krankenkassen zeigen [23]. Bei bestimmten Frakturmustern lässt sich auch mit der Doppelplattenosteosynthese keine ausreichende Stabilität des Tuberculum majus herstellen. In diesen Fällen kann eine dritte Platte als zusätzliche Abstützung dienen und einer posterioren Dislokation entgegenwirken (Abb. 3).

Individualisierte Versorgungen mit 2 oder mehr Osteosyntheseverfahren werden in Deutschland häufiger

Die Nagelosteosynthese kann bei einfachen 2‑Part-Frakturen kopferhaltend eine gute Reposition erreichen. Die funktionellen postoperativen Ergebnisse zwischen Nagel- und Plattenosteosynthese sind vergleichbar [8, 9]. Drei- oder 4‑Part Frakturen hingegen sind schwerer suffizient mithilfe einer Nagelosteosynthese zu versorgen. Biomechanisch zeigen sich vielversprechende Ergebnisse für eine Kombinationsosteosynthese aus Nagel und Platte [28].

Um das Problem des Schrauben-Cut-out und des sekundären Repositionsverlustes zu verbessern, haben zusätzliche Techniken Eingang in die Versorgung am proximalen Humerus gefunden:

Durch die Verwendung von zementaugmentierten Schrauben und die damit erzielte vergrößerte Oberfläche wird das Interface zwischen Osteosynthesematerial und Knochen erhöht. Dies ist insbesondere relevant bei den Schrauben, die sich in einem Knochenareal mit geringer Knochendichte verankern, wie z. B. in der anterioren Region des Humeruskopfes [13]. Werden zementaugmentierte Schrauben verwendet, ist ein besonderes Augenmerk auf die korrekte Schraubenlage zu legen, um einem intraartikulären Zementaustritt vorzubeugen. Ist die Schraubenlage unklar, kann sie zunächst über eine vorherige Kontrastmittelgabe oder einen intraoperativen 3D-Scan verifiziert werden. Mit einer Knochenzementaugmentation ist darüber hinaus das Auffüllen von größeren Defekten möglich, wie sie häufig nach der Reposition von valgisch-impaktierten PHF vorliegen. Die Ergebnisse der verfügbaren Literatur zur Zementaugmentation variieren stark, führen aber insgesamt zu erhöhten Versagenslasten der augmentierten Osteosynthesen [9, 15]. Seit Einführung der Doppelplattenosteosynthese findet die Zementaugmentation am proximalen Humerus im eigenen Vorgehen kaum noch Anwendung.

**Abb. 4:**
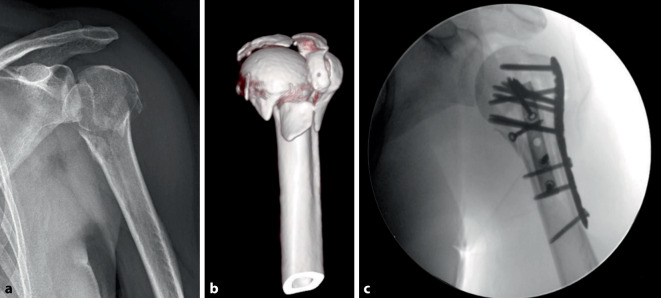
Aufnahmen einer 52-jährigen Patientin mit posteriorer mehrfragmentärer Luxationsfraktur Typ 5 nach Resch et al. [22]. **a** Präoperatives Röntgenbild, **b** Volumenrekonstruktion mithilfe der CT-Bildgebung (Ansicht von anterior), **c** intraoperatives Abschlussbild nach Doppelplattenosteosynthese in Kombination mit autologem Fibula-Graft und freier Schraubenosteosynthese

Ähnlich vielseitig wie die Zementaugmentation ist der Einsatz von verschiedenen Bone-Grafts bei der PHF. Verwendet werden sowohl autogene als auch allogene Materialien. Größere Knochendefekte können mithilfe von Spongiosa, Beckenkammspan oder mit Material aus Femurkopfspenden aufgefüllt werden, benötigen allerdings eine ausreichende kortikale Abstützung. Intramedulläre Verfahren wie ein Fibula-Graft ermöglichen sowohl im Kopf- als auch im Schaftfragment eine Verankerung. Dieses kann der Erleichterung der Reposition dienen sowie eingebrachten Schrauben besseren Halt geben (Abb. 4; [2]).

Sowohl bei der Zementaugmentation als auch bei der Verwendung eines Bone-Grafts muss allerdings ein erschwertes Vorgehen im Verlauf durch verbleibendes Material im Fall einer möglichen Revisionsoperation oder einer späteren Prothesenimplantation berücksichtigt werden.

## Fazit für die Praxis

- Die Platten- und die Doppelplattenosteosynthese sind wichtige Bestandteile der Frakturversorgung am proximalen Humerus.
- Die Wahl der Frakturversorgung erfordert die sorgfältige Betrachtung der morphologischen Frakturmerkale und aller patientenindividuellen Faktoren.
- Bei jungen Patienten sollte die Osteosynthese, wenn möglich, der Prothese vorgezogen werden.
- Die anatomische Rekonstruktion mit Wiederherstellung der Stabilität der medialen Säule senkt die Komplikationsrate durch sekundären Repositionsverlust.
- Zementaugmentationen und Bone-Grafts können eingesetzt werden, um einer Osteosynthese zusätzliche Stabilität zu geben.
